# Maturation and beyond: proteins in the developmental continuum from enamel epithelium to junctional epithelium

**DOI:** 10.3389/fphys.2014.00371

**Published:** 2014-09-25

**Authors:** Bernhard Ganss, Nastaran Abbarin

**Affiliations:** Matrix Dynamics Group, Mineralized Tissue Lab, Faculty of Dentistry, University of TorontoToronto, ON, Canada

**Keywords:** enamel, maturation stage, junctional epithelium, protein identification, SCPP gene cluster

## Abstract

Enamel, covering the surface of teeth, is the hardest substance in mammals. It is designed to last a lifetime in spite of severe environmental challenges. Enamel is formed in a biomineralization process that is essentially divided into secretory and maturation stages. While the molecular events of enamel formation during the secretory stage have been elucidated to some extent, the mechanisms of enamel maturation are less defined, and little is known about the molecules present beyond the maturation stage. Several genes, all located within the secreted calcium-binding phosphoprotein (SCPP) gene cluster, were recently shown to be expressed during the developmental continuum from maturation stage ameloblasts to junctional epithelium (JE). This review introduces four such genes and their protein products, and presents our current state of knowledge on their roles, primarily in enamel formation and JE biology. The discovery of these proteins, and a more detailed analysis of their biological functions, will likely contribute to a more thorough understanding of the molecular mechanisms of enamel maturation and dentogingival attachment.

Enamel is a formidable bioceramic designed to withstand enormous mechanical forces for decades while being subjected to constant changes in temperature, pH and microbial challenges, all that without the ability to regenerate. Thus, the secretory stage of enamel formation has been studied extensively to shed light on the mechanism of enamel formation, which is directly related to three major structural proteins, namely amelogenin (AMEL), ameloblastin (AMBN) and enamelin (ENAM) and two proteases, matrix metalloproteinase (MMP)-20 and kallikrein (KLK)-4. The individual roles of these and other proteins have been studied in great detail *in vitro* and *in vivo* using transgenic mouse models, and we are now beginning to understand the principles of the complex molecular control mechanisms for enamel biomineralization (Moradian-Oldak, 2012). During the following maturation stage the enamel mineral accumulates at the expense of the organic matrix components. Recently some significant progress has been made toward identifying key mechanisms and molecules in this process (Smith, 1998; Simmer et al., 2010; Lacruz et al., 2012a; Damkier et al., 2014). The developmental continuum of ameloblasts beyond the maturation stage, however, has only been described on a histological level, albeit in great detail, and the reader is referred to several excellent reviews on this topic (Schroeder, 1969, 1996; Schroeder and Listgarten, 1997; Bosshardt and Lang, 2005). As teeth erupt into the oral cavity, the reduced enamel epithelium fuses with the oral epithelium and is slowly converted into junctional epithelium (JE) in a coronal-to-apical direction during and after tooth eruption. The JE ultimately provides the “seal” around teeth and is therefore of critical importance to prevent invasion of oral microorganisms, but the molecular composition of the JE attachment apparatus to the mineralized tooth surface remains poorly defined. This short review focuses on four recently identified genes, Amelotin (AMTN), Odontogenic, Ameloblast-Associated (ODAM), Follicular Dendritic Cell Secreted Protein (FDCSP), and Secreted Calcium-binding Phosphoprotein (SCPP), rich in Proline and Glutamine (SCPPPQ1), and briefly summarizes our current state of knowledge regarding their role in enamel and the JE.

## Discovery

AMTN was originally discovered as an enamel-specific gene by differential display analysis of mRNA expression from dental tissues in mice (Iwasaki et al., 2005). An expressed fragment of AMTN was also found as EO-063 in a signal trap screening approach in rat enamel (Moffatt et al., 2006a). ODAM was initially identified as the major protein component in the amyloid deposits of calcifying epithelial odontogenic (Pindborg) tumors (Solomon et al., 2003) and was named APin (for Amyloid in Pindborg tumors). It was also found as EO-009 in the signal trap screening approach mentioned above (Moffatt et al., 2006a) in rat incisor enamel. Due to its high expression in enamel-associated epithelia (Park et al., 2007; Moffatt et al., 2008) it has been re-named ODAM. FDCSP was originally discovered as a secreted protein in follicular dendritic cells (Marshall et al., 2002) and continues to be of great interest in immune cell regulation (Hou et al., 2014). Its relevance to this review comes from its expression in the JE (see below). SCPPPQ1 has been identified as a novel gene in the SCPP gene cluster in mammals (Kawasaki et al., 2011), characterized by its high content of proline and glutamine (PQ). The SCPP and enamel gene clusters are located in close proximity.

## Genomic localization, organization, and protein characteristics

**Figure 1:**
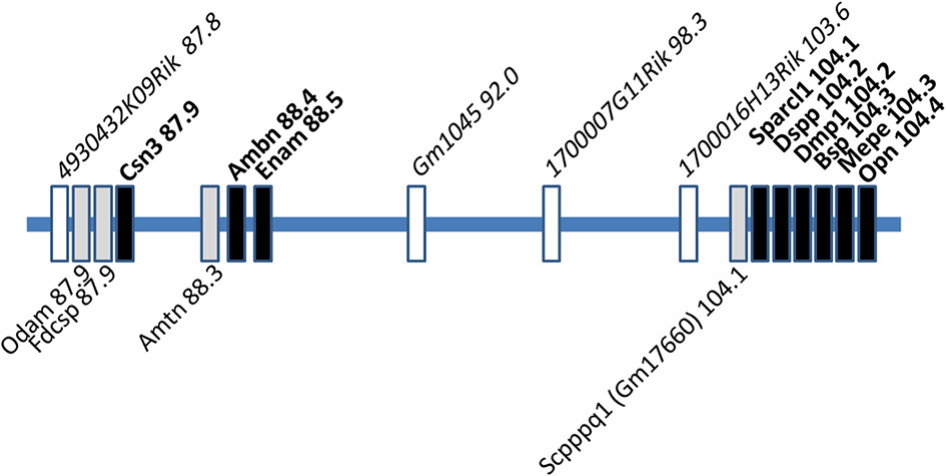
**Schematic presentation of the SCPP gene cluster on the long arm of mouse chromosome 5**. The relative position of the genes is indicated as distance in Mb from the centromere. Well-characterized genes are indicated by black boxes and their names above the central line in bold, uncharacterized genes by white boxes with their names above the central line in italics. The four genes reviewed here are shown below the central line.

The genomic location of *AMTN, ODAM, FDCSP*, and *SCPPPQ1* contains clusters for genes involved in milk (e.g., caseins), saliva (e.g., statherins), enamel (e.g., *AMBN, ENAM*), and bone (small integrin-binding ligand N-linked glycoproteins, SIBLING family) and their evolutionary and functional aspects have been extensively reviewed (Huq et al., 2005; Kawasaki and Weiss, 2008; Kawasaki, 2011; Kawasaki et al., 2011; Staines et al., 2012). The more recently discovered *AMTN, ODAM, FDCSP*, and *SCPPPQ1* genes are located within this cluster in the proximal-to-distal sequence *ODAM, FDCSP, AMTN, SCPPPQ1* and this sequence appears to be conserved in mammalian genomes. Figure 1 shows a schematic map of the mouse chromosome 5 and the relative location of genes of interest. Table 1 summarizes genomic organization, protein characteristics, expression and possible functions for the four proteins discussed here. Data for the human orthologs are similar unless indicated otherwise.

**Table 1:** **Summary of genes of interest, their designation, genomic organization, protein characteristics expression profile, and possible functions**.

**Gene name**	**Odontogenic, ameloblast-associated**	**Follicular dendritic cell secreted protein**	**Amelotin**	**Secretory calcium-binding phosphoprotein, rich in proline and glutamine**
Abbreviation and alternate designations	ODAM (APin)	FDCSP (C4orf7; human)	AMTN	SCPPPQ1 (Gm17660; mouse)
Genomic organization	12 exons	5 exons	9 exons	10 exons
Protein characteristics	28.3 kDa, pI 4.83	8.0 kDa, pI 4.46	20.4 kDa, pI 5.88	8.3 kDa, pI 4.68
	PQ rich	P rich	PLTQG rich	PL rich
	P-Ser, p-Thr	P-Ser, p-Thr	P-Ser, p-Thr, p-Tyr	P-Ser
	O-gly	O-gly	O-gly	
Predominant expression	Maturation ameloblasts, junctional epithelium	Tonsils, lymph nodes, junctional epithelium	Maturation ameloblasts, junctional epithelium	Maturation stage ameloblasts
	Nasal and salivary glands, oral epithelial tumors			
Possible functions	Multifunctional matricellular protein, Regulation of MMP-20	Regulation of inflammation in the JE	Induction of mineralization, cell attachment	Unknown

*Note that phosphorylations of serine (p-Ser), threonine (p-Thr), and tyrosine (p-Tyr) as well as O-glycosylations are predicted in silico, not experimentally proven*.

All four proteins contain an N-terminal signal sequence and have either been shown (Iwasaki et al., 2005; Moffatt et al., 2008) or are expected to be secreted. The *Amtn* gene consists of nine exons and the mature, secreted AMTN protein (after cleavage of the N-terminal signal sequence) is 20.4 kDa in size with a pI of 5.88. It is rich in proline, leucine, threonine, glutamine, and glycine and is predicted to be modified by serine, threonine and tyrosine phosphorylation and O-glycosylation. AMTN expression seems to be restricted to maturation stage ameloblasts and the JE, although the application of increasingly sensitive techniques has shown *Amtn* transcripts in calvaria-derived osteoblasts (Atsawasuwan et al., 2013). Whether this low level expression has any functional implication *in vivo* remains to be determined. The *Odam* gene consists of 12 exons and the protein is 28.3 kDa in size with a pI of 4.83. It is rich in glutamine and proline and predicted to be phosphorylated at multiple serine and threonine residues and O-glycosylated at multiple positions. ODAM expression is highest in maturation stage ameloblasts and JE, but present in other tissues including nasal and salivary glands (Moffatt et al., 2008), indicating a broader biological role. The *Fdcsp* gene consists of five exons and its product is a small (8.0 kDa), very proline-rich polypeptide with a pI of 4.46. It is predicted to be phosphorylated at serine and threonine residues, and possibly O-glycosylated. FDCSP is predominantly expressed in tissues related to the immune system including tonsils and lymph nodes, but also in tooth supporting structures such as the JE (Shinomura et al., 2008) and periodontal ligament (Nakamura et al., 2005). Its expression in ameloblasts has not been reported. The *SCPPPQ1* gene (annotated as *Gm17660* in the mouse) consists of 10 exons and the protein is 8.3 kDa in size with a pI of 4.68, rich in proline and leucine. The protein is predicted to be phosphorylated at two serine residues at the N-terminus, but not glycosylated. All four proteins are predicted by *in silico* analyses to be intrinsically disordered, a characteristic shared with other known mineralization regulators in the SCPP gene cluster. None of the four proteins contain integrin-binding “RGD” motifs or N-glycosylation sites.

## Roles in enamel formation

AMTN has been identified by differential display analysis of mRNA expression from dental tissues (Iwasaki et al., 2005) and is, based on lack of expression in other tissues, considered to be specific to the ameloblast lineage. Evolutionary analyses have provided further evidence for AMTN as an enamel-specific protein (Gasse et al., 2012). The expression of mouse amelotin was found by several independent groups to be rapidly and dramatically upregulated in ameloblasts at the transition from secretory to maturation stage, and this expression profile is very different from that of the more established enamel proteins AMEL, AMBN, and ENAM (Moffatt et al., 2006b; Trueb et al., 2007; Somogyi-Ganss et al., 2012). In contrast, a single report using non-affinity-purified antibodies has shown Amtn expression in secretory stage enamel matrix of the mouse (Gao et al., 2010). The secreted AMTN protein accumulates at the interface between cells and enamel mineral and appears to be part of the specialized basal lamina-like layer that reappears at the onset of the maturation stage (Dos Santos Neves et al., 2012; Somogyi-Ganss et al., 2012). This restricted and specific localization of AMTN suggests functional roles in cell adhesion and/or surface enamel mineralization. Since AMTN can form multimeric aggregates in solution (Holcroft and Ganss, 2011) it is also possible that it is part of a basal lamina-like structure that may be involved in transport control in and out of the maturing enamel. We have described a simple cell adhesion experiment where bacterially produced, recombinant (and therefore lacking post-translational modifications) AMTN did not mediate any cell attachment (Somogyi-Ganss et al., 2012). However, since AMTN is predicted to contain P-serine and potentially O-glycosylations, these modifications may alter the adhesive properties of the protein. It may also be possible that AMTN requires other interacting proteins to mediate cell adhesion, and in this context it is particularly interesting that AMTN has been shown to interact with ODAM (Holcroft and Ganss, 2011). More detailed protein interaction studies are underway to determine whether other proteins bind to AMTN and/or ODAM in a multimeric complex that may mediate cell attachment. It is currently not known whether AMTN plays any role in controlling the transport of ions and/or protein fragments in and out of the maturing enamel. Preliminary experiments in our lab have failed to detect any significant differences in staining pattern of incisal enamel with pH sensitive dyes between wild type and AMTN KO mice. This indicates that the naturally occurring proton transport during enamel maturation is not fundamentally altered. It would be interesting to probe the potential role of AMTN in ion transport by comparing the distribution of fluoride that is supplied in the drinking water (Lyaruu et al., 2014) in wild type and AMTN-deficient mice in light of dental fluorosis as a clinical problem. The expression of AMTN coincides with the establishment of the dense, highly mineralized, aprismatic surface enamel layer (Moinichen et al., 1996) and it is conceivable that the protein is involved in the establishment of this layer. We have recently reported transgenic mice engineered to overexpress AMTN under the AMEL gene promoter (Lacruz et al., 2012b). These mice express AMTN not only at higher amounts, but also earlier during amelogenesis and as a result show a hypoplastic yet densely mineralized irregular enamel layer that does not show any organized rod and interrod microstructure, indicating that AMTN may have an accelerating role in the controlled mineralization of hydroxyapatite. We have recently developed AMTN-deficient animals, and it will be interesting to determine their phenotype with particular attention to the mineralization of the surface enamel mineral structure and density.

ODAM is expressed in a pattern very similar to AMTN during enamel formation. The first published report that indicated an involvement of ODAM in enamel formation showed high expression during amelogenesis in the rat incisor (Park et al., 2007) using immunohistochemistry and *in situ* hybridization. The authors further postulated that ODAM could exert its function during enamel formation and mineralization by modulating the expression of the enamel protease MMP-20. The onset of ODAM expression is slightly earlier (late secretory stage) than AMTN, but both proteins are localized in the basal lamina-like layer at the ameloblast-enamel interface. There appears to be a subtle difference in ODAM/AMTN localization with ODAM closer to the cell surface and AMTN closer to the enamel surface (Dos Santos Neves et al., 2012), but whether this bears any functional significance is not known. ODAM KO mice have not been described in the literature to date. Further studies on the molecular mechanism of ODAM-mediated regulation of enamel mineralization have postulated that an intracellular form of ODAM is phosphorylated by the bone morphogenetic protein receptor type IB (BMPR-IB)-mediated action of BMP-2 and thus modulates the signaling pathways involved in ameloblast differentiation (Lee et al., 2012a). The same group has further shown that intracellular ODAM cooperates with the runt domain transcription factor Runx2 and thus regulates the expression of MMP-20. This modulation of MMP-20 by ODAM was interpreted as a mechanism by which enamel matrix maturation is regulated by ODAM (Lee et al., 2010). While the notion of an intracellular form of ODAM is interesting and not unprecedented—as shown for the SIBLING protein osteopontin (OPN) (Zohar et al., 1997, 2000)—it remains to be explained how and why the ODAM protein is retained or accumulates in the cytoplasm and the nucleus of ameloblasts *in vivo* and in culture. Presumably it is the intracellular/nuclear form of ODAM that is involved in MMP-20 regulation, but details of this process also remain to be determined. These findings contrast the identification of ODAM from rat ameloblasts by a signal trap screening approach (Moffatt et al., 2006a), which is designed to target secreted proteins. Interestingly, a direct inductive effect on dentin mineralization was observed when recombinant ODAM protein was applied to odontoblast cells in culture and in a dental pulp capping experiments in rats *in vivo* (Yang et al., 2010), but whether a similar effect would be observed in enamel is currently unexplored.

The expression profiles of SCPPPQ1 and FDCSP during enamel formation have not yet been detailed in published work, but SCPPPQ1 has been identified as EO-463 by screening for secreted proteins in rat enamel (Moffatt et al., 2006a) and the expression of SCPPPQ1 has been demonstrated in the basal lamina-like layer of late maturation stage ameloblasts (Wazen et al., 2013). Knockout animals for FDCSP have been developed (Hou et al., 2014), but no phenotype related to teeth has been reported, even though FDCSP apparently binds to hydroxyapatite (Shinomura et al., 2008). A knockout for SCPPPQ1 has not been published. Thus, the expression profiles and potential role(s) for these two proteins in enamel formation remain to be elucidated.

## Roles in the junctional epithelium

AMTN protein is continuously detected at the ameloblast-enamel interface from the maturation through the reduced enamel epithelium to the JE in mice (Somogyi-Ganss et al., 2012) and rats (Moffatt et al., 2006b; Nishio et al., 2013). The localization of AMTN in the JE is restricted to the cell/mineral interface, while ODAM is localized in a pericellular fashion in the JE. In this context it is interesting to note that the presence of AMTN during formation of the primary JE has only been detected by immunohistochemistry, but not by *in situ* hybridization, which either indicates insufficient sensitivity of the technique to detect low abundance Amtn mRNA transcripts, or that the AMTN protein found in the JE is residual from production by the reduced enamel epithelium (Sawada et al., 2013). Regardless, both AMTN and ODAM are re-expressed in the JE after gingivectomy and regain their normal expression pattern after the gingival tissue has regenerated (Nishio et al., 2010a). Notably, ODAM expression is induced in epithelial rests of Malassez (ERM) after experimental periodontal detachment and ODAM is re-expressed during orthodontic tooth movement (Nishio et al., 2010b; Jue et al., 2013), indicating that ODAM may play a role in periodontal regeneration. However, the work to date is largely descriptive and functional studies have not been published. Aside from its role in the immune system, a growing body of literature indicates the involvement of FDCSP in the dentogingival attachment apparatus. The specific pericellular expression of FDCSP in the PDL (Nakamura et al., 2005) and the JE (Shinomura et al., 2008) has been described earlier, followed by the observation that periodontal pathogen-derived lipopolysaccharide (LPS) leads to rapid loss of FDCSP expression in an experimental rat model (Oshiro et al., 2012). Exposure of periodontal ligament cells to FDCSP in culture resulted in increased cell proliferation and suppression of mineralization-associated gene expression (Wei et al., 2011), possibly indicating a role for FDCSP in maintaining these cells in a fibroblastic state that allows them to rapidly respond to traumatic events. Overexpression of FDCSP in PDL cells by viral transfection also reduced the level of osteogenic gene expression (Xiang et al., 2013a,b), presumably stabilizing a fibroblastic phenotype. In a ligature-induced model of periodontitis in rats the expression of FDCSP was significantly decreased during the establishment of periodontal attachment loss, while interleukin 17 (IL-17) and the RANKL/OPG ratio were up regulated, indicating a state of acute inflammation and increased bone resorption, respectively. This suggests a possible role for FDCSP in mediating the inflammatory responses to periodontal pathogens in the JE, and ties back to the documented immunmodulatory role of FDCSP (Al-Alwan et al., 2007; Hou et al., 2014).

A recently published *in vitro* model system for junctional, sulcular and gingival epithelium formation has used ODAM and FDCSP expression to delineate the JE (Dabija-Wolter et al., 2013). Further support for a potential role of the four genes described here in periodontal health was provided in a recent report that linked regions in the enamel gene cluster to periodontitis susceptibility by QTL analysis (Shusterman et al., 2013).

## Roles in tumors

In addition to the potential functions in enamel and the JE, there has been some interest in the expression of these enamel gene cluster proteins in cancers. For ODAM this was expected, since it was initially discovered in Pindborg tumors (Solomon et al., 2003; Murphy et al., 2008). ODAM has further been found in other types of lung and breast tumors of epithelial origin and has been suggested as a prognostic marker for such neoplasms (Kestler et al., 2008), particularly human breast cancer (Siddiqui et al., 2009). Other descriptive work focusing on the expression of enamel proteins has confirmed the expression of ODAM and AMTN in certain types of odontogenic tumors (Ren et al., 2011; Crivelini et al., 2012), often associated with sites of mineral deposition (Stolf et al., 2013). ODAM expression has further been described in odontoblasts, osteoblasts and various cancer cells (Lee et al., 2012b). The first study looking at functional aspects of the expression of ODAM in tumors has shown that it can inhibit tumorigenic characteristics in the human breast cancer cell line MDA-MB-231 *in vitro* and the transplantation of ODAM-expressing tumor cells into mice lead to significantly reduced tumor growth and their inability to metastasize, compared to control cells that did not express ODAM (Kestler et al., 2011). Further dissection of the molecular pathways affected by ODAM revealed that it can act through elevation of the tumor suppressor phosphatase and tensin homolog (PTEN) and inhibition of the apoptosis-blocking PI3 kinase/AKT pathway (Foster et al., 2013). One published report indicates that FDCSP (designated C4orf7 at the time) can modulate cytoskeletal actin dynamics, thereby promoting migration and invasion of ovarian cancer cells (Wang et al., 2010)

## Other genes within the SCPP gene cluster

In addition to the enamel and SIBLING gene cluster, which contain genes involved in mineralized tissue formation, other calcium binding phosphoproteins are found in this genomic region. These include the casein genes, salivary protein genes (statherin etc.), but also a number of uncharacterized annotated genes. The issue of poor and incomplete annotation makes it difficult to predict the existence and function of additional genes in this cluster. For example, the mouse genome contains a gene annotated as 4930432K09Rik in the ensembl genome browser (http://www.ensembl.org), which codes for a predicted protein of 344 and 352 amino acids from two alternatively spliced transcripts, just upstream of the ODAM gene. A presumptive human ortholog, annotated as C4orf40, has significant sequence similarity with parts of 4930432K09Rik, but encodes a protein of only 219 amino acids. Both C4orf40 and 4930432K09Rik predicted proteins contain a well-conserved N-terminal signal sequence, are mildly acidic (pI 4.82 and 5.30, respectively), proline-rich and contain multiple predicted serine, threonine and tyrosine phosphorylation as well as O-glycosylation sites. Similarly, other uncharacterized, protein-coding genes are currently annotated in the human genome as C4orf26, C4orf22, C4orf36, and in the mouse genome as Gm1045, 1700007G11Rik, and 1700016H13Rik, all within the enamel/SIBLING gene cluster. C4orf26 has recently been described as a candidate gene for *amelogenesis imperfecta*, and the protein apparently possesses hydroxyapatite nucleation and growth activity (Parry et al., 2012). Interestingly, the FDCSP gene is not annotated as such in the mouse genome in both the ensembl (http://www.ensembl.org/index.html) and UCSC (https://genome.ucsc.edu/) genome browsers, even though the genomic organization (Marshall et al., 2002) and even knockout animals (Hou et al., 2014) have been described. Clearly, much work remains to be done to discover and understand the biology of additional proteins coded by other genes in the SCPP gene cluster.

In conclusion, the SCPP gene cluster contains multiple genes that have been studied to various extents in the context of mineralized tissue. AMTN, ODAM, FDCSP, and SCPPPQ1 have been added to the more established enamel and SIBLING genes, and other genes await characterization. The expression of AMTN, ODAM, and FDCSP has emphasized the developmental continuum from the reduced enamel epithelium to the JE on a molecular level and may become instrumental in understanding the molecular details of the dentogingival attachment apparatus.

### Conflict of interest statement

The authors declare that the research was conducted in the absence of any commercial or financial relationships that could be construed as a potential conflict of interest.
